# Editorial: Pharmacometabolomics: biomarker discovery, precision medicine, technical advances, perspectives and future applications in respiratory diseases

**DOI:** 10.3389/fmolb.2023.1268001

**Published:** 2023-08-30

**Authors:** Alessia Vignoli, Panteleimon Takis, Paolo Montuschi

**Affiliations:** ^1^ Magnetic Resonance Center (CERM), University of Florence, Sesto Fiorentino, Italy; ^2^ Department of Chemistry “Ugo Schiff”, University of Florence, Sesto Fiorentino, Italy; ^3^ Consorzio Interuniversitario Risonanze Magnetiche MetalloProteine (CIRMMP), Sesto Fiorentino, Italy; ^4^ Department of Metabolism, Digestion and Reproduction, National Phenome Centre, Imperial College, London, United Kingdom; ^5^ Faculty of Medicine, Imperial College, National Heart and Lung Institute, London, United Kingdom; ^6^ Pharmacology, Faculty of Medicine, Catholic University of the Sacred Heart, Roma, Italy

**Keywords:** metabolomics, pharmacometabolomics, personalized medicine, respiratory diseases, biomarker discovery, targeted pharmacotherapies, molecular phenotyping

To clarify the molecular bases of the heterogeneity of respiratory diseases and develop personalized pharmacotherapies is one of the main priorities in respiratory medicine research. Metabolomics is a unique tool for exploring the pathophysiology of respiratory diseases, enabling us to identify potential targets for the research and development of new drugs, and discover new biomarkers for monitoring disease and the effects of therapeutic intervention. The five articles published in this Research Topic focus on pharmacometabolomics and its potential implications for biomarker discovery and personalized medicine, with discussions of technical advances, perspectives, and anticipated applications in respiratory diseases.

The severity of obstructive sleep apnea syndrome (OSAS), a major sleep-related breathing disorder with a prevalence of 9%–38%, is associated with an increased risk of comorbidities, including chronic kidney disease, chronic obstructive pulmonary disease (COPD) and cardiovascular diseases. Aiming at discovering plasma biomarkers measured by untargeted gas chromatography-mass spectrometry in severe OSA individuals, Mohit et al. compared the metabolic profiles of severe OSAS participants with those of healthy control participants in a cross-sectional observational study. In persons with OSAS, metabolomics revealed changes in plasma levels of selected metabolites compared with healthy control subjects. As an example, plasma concentrations of serotonin, indoxyl sulfate, and 5-aminolevulinic acid were elevated, while those of tryptophan, glutamic acid and proline were reduced in persons with severe OSAS. Enrichment and pathway analysis also showed significant changes in individuals with severe OSAS compared with healthy individuals. Among various pathways involved, pathways related to amino acid metabolism, ascorbate and aldarate metabolism and glycerolipid metabolism were the most significantly affected by OSAS. If externally validated, these findings might provide potential plasma biomarkers for OSAS.

Yu et al. present a systematic review that includes a meta-analysis of 10 randomized clinical trials with a total population of 1,751 individuals. This study assesses the efficacy and safety of p38 mitogen-activated protein kinase (MAPK) inhibition as a potential therapeutic strategy for COPD, reporting no difference in the efficacy and safety between p38 MAPK inhibitors and placebo. However, the limited number of studies included and the heterogeneity from combining various p38 MAPK inhibitors as a whole preclude definitive conclusion on the assessment of this potential pharmacotherapy for COPD.

Up-regulation of the transforming growth factor *β* (TGFβ) pathway(s) plays a primary role in pulmonary vascular remodeling. To investigate new potential therapeutic strategies for pulmonary arterial hypertension based on the targeting of TGFβ signaling, Morales-Cano et al. studied the effects of 5Z-7-oxozeaenol, an inhibitor of TGFβ-activated kinase 1 (TAK-1), alone or in combination with riociguat, a vasodilator drug, in an *in vivo* rat experimental model of pulmonary arterial hypertension. Using nuclear magnetic resonance spectroscopy, *in vitro* metabolic profiling of the rat right ventricle, lung tissues, and pulmonary artery, smooth muscle cell extracts were also performed. The results showed that 5Z-7-oxozeaenol reduced pulmonary vascular remodeling and the metabolic shifts of glucose and phosphocholine in the right ventricle, without any effects on pulmonary artery pressure or right ventricle hypertrophy. The combination of 5Z-7-oxozeaenol and riociguat had an additive effect on pulmonary vascular remodeling and induced a significant metabolic effect over taurine, amino acids, glycolysis, and tricarboxylic acid cycle metabolism via glycine-serine-threonine metabolism. However, it did not improve the effects induced by riociguat alone on pulmonary pressure or right ventricle remodeling. This study shows that inhibition of TAK-1 elicits antiproliferative effects and its addition to short-term vasodilator therapy increases the beneficial effects on pulmonary vascular remodeling and right ventricle metabolic reprogramming in an experimental model of pulmonary arterial hypertension.

The review article by Meng et al. provides an overview of pharmacotherapies for acute respiratory distress syndrome, including conventional drugs, natural medicine therapy, and nanomedicine. In particular, they focus on nanoparticles as drug delivery vehicles, which have been extensively studied in the treatment of acute respiratory distress syndrome, and discuss nanomedicine, which has great therapeutic potential for acute respiratory distress syndrome.

Nuclear factor erythroid 2-related factor 2 (Nrf2) is a transcription factor that interacts with multiple signaling pathways, regulating the activity of various oxidases, including NADPH oxidase, nitric oxide synthases, xanthine oxidase, and cytochrome P450 isoenzymes, related to inflammation and apoptosis, and exhibits antioxidant and anti-inflammatory activity in acute lung injury.

Another review article by Luan et al. summarizes evidence supporting the protective effects of natural pharmaceutical components, including polyphenols, flavonoids, terpenoids, alkaloids, and polysaccharides on acute lung injury by activation of the Nrf2 signaling pathway.

In summary, the articles collected in this Research Topic showcase the potential translational value of metabolomics. Metabolomics and other -omics data could be successfully integrated by artificial intelligence and machine learning algorithms, with the aim of implementing personalized pharmacotherapies in respiratory medicine as well as the development of targeted therapies.

